# Feasibility and relatability of cultural adaptation amongst conflict-affected populations

**DOI:** 10.1016/j.jmh.2022.100134

**Published:** 2022-09-24

**Authors:** Lyla Schwartz, Donja Brunner, Eva Unternährer, Christina Stadler

**Affiliations:** Universitare Psychiatrische Kliniken, University of Basel, 4002 Basel, Switzerland

## Abstract

Cultural aspects, such as cultural values and specific stressors, need to be considered in intervention development of care.^1^ One study looked at the relevance and necessity of cultural adaptation in mental health interventions, and the results were conclusive. They found that for populations the intervention was not designed for, treatment was less effective than for the target population.^2^ Existing mental health programs well established in developed countries, with a low-threshold for quality care and a flexible delivery of the care, have shown to be less effective for marginalized populations and refugees, where linguistic and cultural barriers still exist. These barriers include limited understanding of treatment, a lack of knowledge on the healthcare systems in host countries, and the lack of interpreters or programs available in their native language.^3^ A promising approach for culturally-informed treatment are short skills-training interventions, designed to help participants better cope with stress and build up resilience to successfully deal with new challenges. START NOW is an evidence-based integrative skills training program and an effective treatment for vulnerable adolescents in demanding situations. We designed a total of eleven characters and fifthteen storylines that reflect the negative experiences commonly experienced by the target beneficiaries, and then conducted content testing to identify the most relatable among them. By conducting content testing, we can culturally adapt the START NOW program to meet the needs of Persian and Afghans migrants, informed by the very community that the program seeks to help. The results identified the six most relatable characters and twelve most relatable storylines, which will go on to feature in START NOW intervention sessions, and improve outcomes in testing the adapted program's efficacy with participants in Switzerland. This study provided a greater understanding of the challenges and emotions experienced by Persian and Afghan migrants, and enables us to design an effective, culturally-relevant treatment to address symptoms of poor mental health amongst this population. We can now replicate this process when adapting evidence-based treatments for other populations in need.

## Highlights

• Migrants in Switzerland are faced with disproportionate barriers to mental health services and access to effective interventions is limited.
• START NOW is an evidence-based, integrative skills training program, incorporating core features of resilience, such as effective emotion and stress regulation, self-efficacy and cognitive reappraisal.
• Content testing was conducted with the feasibility and reliability of characters and storylines scored on a Likert scale by participants.
• There is a clear correlation between reporting high levels of distress and higher relatability to characters.
• This higher rate of relatability should contribute towards a greater reduction in symptoms of depression, anxiety and stress in participants.

## Introduction

Afghan migrants currently make up a large portion of migrants arriving and claiming asylum in Europe.^4^ They are suffering from the impacts of forty years of war and have arguably, some of the lowest accessibility to care of any group. They face linguistic and cultural barriers; the country's system of trained interpreters is underfunded and unregulated, producing long waiting times for mental health treatment and an insufficient quality of care when available.^5^ A study of 78 asylum seekers living in Zurich, over a 12 month period, found that the population sought treatment on average twice as much as a national resident, yet incurred higher treatment costs and a notable lack of specific treatment.^6^ It was found that health professionals tended to under-diagnose them and not provide the appropriate treatment, because of linguistic barriers as well as others.^7^ In addition, existing mental health programs tend to be less effective or even ineffective for refugee populations.^8^ Yet there is a need for low-intensity interventions that can reach high numbers of patients, that have a low-threshold for care and flexible delivery of the care. Such a program must surmount barriers such as poor understanding of treatment, poor knowledge of healthcare systems in host countries, and the lack of interpreters or programs available in multiple languages.^9^ Given evidence that mental health interventions work much better for the populations for which they are designed,^10^ it must reflect cultural values and specific stressors particular to the target population.^11^ Researchers have begun to look at the process of culturally adapting scalable psychological interventions for migrants^12^. However, no randomised controlled trial has yet investigated the efficacy of an adapted skills training for migrants coming from Afghanistan, and indeed, culturally sensitive programs are rare. Thus, by culturally adapting short skills training interventions for this population, we can mitigate the accessibility and affordability of mental health treatment. This project addresses both of this gap by investigating the efficacy of a contextualised mental health intervention.

START NOW is an evidence-based cognitive behavior therapy (CBT) program developed by Prof. R. Trestman at the University of Connecticut^13^ and adapted by Prof. Dr. C. Stadler at the University of Basel.^14^ The program is an evidence-based, manual-guided, gender-specific skills training that combines aspects of cognitive behavioral therapy (CBT), motivational interviewing (MI), dialectical behavior therapy (DBT), Acceptance and Commitment Therapy (ACT) and trauma-sensitive care.^15^ The program is an evidence-based integrative skills training and an effective treatment for vulnerable adolescents in demanding situations. The existing START NOW training includes a manual with detailed instructions for group facilitation and a workbook for participants^16^.

The overall goal of this project is to culturally adapt the START NOW skills training to meet the needs of adolescent and young adult Persian / Afghan migrants. Following adaptation, the program should retain its high quality as a means to increase mental wellbeing and to reduce symptoms of depression, anxiety, stress, persistent sadness, feelings of guilt and shame, and agitation in the target population.

START NOW is a manual-based program that features detailed instructions for group facilitation and a participant workbook with eleven characters between 14 and 24 years and fifthteen storylines. The new storylines describe negative experiences migrants may face and the difficulties of managing their emotional responses. The project involves (i) adapting these characters and 15 storylines such that the characters are new migrants whose visual representations suggest they may be Persian or Afghan men and women, and (ii) testing their relatability in young adult Persian / Afghan migrants using an online survey. In addition, we were interested (iii) whether the developed story lines are also appropriate in migrants coming not from Afghanistan.

By conducting this content testing, our aim was to gather feedback on characters and storylines, to identify which characters and storylines are most relatable to Persian / Afghan migrants, and to then use this feedback to select the most relatable to feature in the culturally adapted START NOW training.

## Method

### Participants and procedure

For this cross-sectional online study, we contacted young migrants in Switzerland and in other host countries through the institutes they reside in and via online platforms, including public groups on Facebook and Linkedin, from February 2022 to March 2022. We specified that migrants who speak at least some Farsi and whose nationality is Afghan or Iranian and are aged 14 to 40 were eligible to participate. Ultimately we recruited 291 migrants from Afghanistan (n=223) and Iran (n=68) who completed sufficient questions in the survey for the study to be usable. The sample included 81 women (27.8%) and 207 men (71.1%; unknown: n=3, 1,0%), with a mean age of 28.25 years (SD=5.26 years). Most of the sample, n=163 were currently living in Switzerland. There were 39 living in Afghanistan, who were internally-displaced. The remainder lived in a range of different countries (n=42), the United States (n=28) and Germany (n=17). Two omitted where they were living.

### Measures

Participants completed an online survey on Qualtrics featuring the adapted characters and storylines besides some general demographic questions. The survey was available in English or Farsi, and was adapted for use on any device with an internet connection.

All participants were required to provide written informed consent for anonymous study participation prior to beginning the survey. Since this was a fully anonymized study, it did require authorization according to the Human Research Act or a Swiss ethics committee.

The survey's introduction consisted of a description of the study including the purpose of the content testing and they were asked to provide consent to participate. Demographic questions consisted of questions relating to age, gender (self-identified male or female), language abilities (Farsi), country of origin, whether they currently resided in Switzerland, what year they arrived in Switzerland (if applicable), and whether they want to remain in Switzerland (if applicable), and to what extent they currently experience psychological distress measured on a Likert scale from 0 (no stress) to 5 (lots of stress).

The participants then reviewed the eleven characters’ background, immigration experience, and current circumstance, and were shown an illustration of a man or woman with Persian / Afghan features and dress. No names were given for the characters to avoid participants feeling an emotional connection to the characters based off of their names, characters were instead identified by a feeling such as “loneliness,” “guilt”, a number and an initial. Participants were asked to rate the character on a Likert scale from 0 (not relatable at all) to 5 (very relatable).

Last, participants were presented with 15 storylines. The storylines described a negative event commonly experienced by migrants, and the characters’ emotional response. Each of the eleven characters were featured in one or two of the storylines, depending on which emotion fit. Again, participants were asked to rate the character from 0 (not relatable at all) to 5 (very relatable).

The survey concluded with an option for participants to leave additional feedback about the content testing process.

### Content; Characters and Storylines

#### Storylines

1. Lives Lost (character A)

A is sitting on his bed looking at pictures of him and his best friend on his phone.

Peers come to his door and invite him to join them for a walk, A declines.

A can't eat or sleep, he is having flashbacks to his friend passing away and doesn't know how to feel OK again.

2. Not Making Friends (character B)

B is walking with two female family members, they see a small group of women they know from afar, they wave.

The women meet and start chatting, B is not talking with anyone.

B tries to join the conversation but she is ignored.

3. All Alone (character C)

C is on video call with her family back home, with her daughter on her lap. Her family are smiling and waving at the baby.

C hangs up the phone.

She heads to the window and looks outside, she can see a group of women with babies, all chatting. She feels so alone, and doesn't have anyone for her daughter to play with.

4. Awaiting Asylum (character D)

D's two friends have had their asylum status approved.

The two friends are packing their suitcases as they prepare to move, they are smiling and laughing. D is constantly checking his phone for a decision on his asylum case.

D can't eat or sleep because of how stressed he is, he stopped playing football each week or keeping in contact with his family too much.

5. All These Rules (character E)

E is hanging outside with his friends, they are laughing and kicking a ball around.

Security at the asylum centre calls them inside, telling them its past curfew.

The group head inside but E is getting pissed off, he violently kicks the football against the building's front door. He is frustrated that he has to follow so many rules.

6. High Expectations (character F)

F is waiting in line to buy a sandwich. He receives text messages from his family asking him to help them by sending some money.

F looks down into his hand, he is holding just a few coins.

He leaves the queue for the sandwich empty handed, he is stressed about having so little money, and the constant pressure to support his family.

7. Lost Dreams (character G)

G is sitting in a cafe and sees a group of students walking by holding textbooks and smiling.

She has a flashback to sitting in class with her peers in Afghanistan, enjoying her studies.

G is bored with her life now, she is disappointed she had to leave Afghanistan and struggles to have hope for the future.

8. Left Behind (character H)

H is on video call with his parents and brothers, they are all chatting.

His brother asks when H will bring him to Europe, H doesn't know how to respond. His family are reliant on his bringing them to Europe or financially supporting them. He often feels guilty and under a lot of pressure to support his family financially.

9. Odd One Out (character I)

I is riding the bus alone, she sees a group of girls her age pointing and laughing at her.

She sees her reflection in the bus window, she wears the hijab and looks different to all the other girls. I quickly hurries off the bus. She always feels like she doesn't fit in in Switzerland.

10. Noone to Help (character J)

J receives a negative decision on his asylum application.

J begins to call lawyers, the first lawyer hangs up on him when he doesn't speak German, the second says he can't help without payment.

J asks his peers for advice, and no one can give him any help. He feels hopeless and like there is no one to support him.

11. New Life, New Language (character K)

K is sitting in a German class.

The teacher is saying a lot of new words and K can't keep up. Everyone else starts practising the new German words, K is lost and doesn't know what to say.

K tells herself she is stupid and will never learn German. She feels disappointed in herself and frustrated.

12. Just Like Them (character L)

L in a supermarket looking at the snack aisle, peers around the same age are also in the store.

L watches as they put snacks into their pockets without paying. L tries the same, but is stopped by security. She just wants to fit in, she is embarrassed and scared to tell her parents.

13. Fitting In (character M)

M has made some friends with local Swiss boys, he meets them to hang out.

The group of boys buy some cigarettes and try them in the park. Some other teenagers from the asylum centre see them and tell M's parents. His family are very angry with him.

He feels like he has no control over his life, and he just wants to grow up and have his independence.

14. Choosing Not to Pray (character N)

N doesn't want to pray every time, he likes to read books or meditate to feel calm.

The other boys in the asylum centre say rude things about him and bully him about this. N just wants to be accepted.

15. Struggle to Find Work (character O)

O gets dressed and ready to go out and apply for jobs.

O walks into a business and hands out his CV, he is rejected straight away, but he watches as a Swiss man walks into the same building and has his CV taken by the manager. O feels judged and upset, he is struggling to support himself or his family and is frustrated he is not given a chance.

### Statistical analysis

Differences in relatability between gender and nationality was investigated by using independent t-tests. In addition, to assess the relation between relatability (person/storyline) and psychosocial distress and age Spearman's Rank correlation coefficients were calculated.

And relatability between gender and nationality using independent sample t-tests, and investigated associations with age using Spearman's Rank correlation coefficients. Finally, we also examined associations of distress and relatability using Pearson correlations.

Relatability ratings of characters and storylines were measured on a Likert scale from 0 (not relatable at all) to 5 (very relatable). Analysis found overall, characters were more relatable than storylines, with the mean scores of characters sitting between 1.74 and 2.35, in comparison to between 1.01 and 2.56 for storylines. Each score was analysed against gender and nationality, and the difference between Women and Men, and Afghan and Iranian, calculated. In addition, the correlation between age and score, and psychosocial distress and score, was also calculated.

## Results

### Demographics and psychological distress

Analyses indicated that distress was not correlated with age (rho=-0.02; p=0.69) and there was no difference between female and male migrants (t(132.3)=0.798; p=.426), but that there was a difference according to nationality (t(117.6) = 2.493; p=.014). Iranians (M=3.34, SD=1.41) were experiencing higher levels of distress than Afghans (M=2.84, SD=1.51).

### Character relatability

We found a significant correlation between age and relatability of character #5; and of distress with relatability of all characters (Table 1). Across all participants, we found that the highest rated characters were #6, #2, and #1 (Table 1). Compared with men, women related significantly stronger with #6 (Table 1). Moreover, compared with Iranians, Afghans related significantly stronger with #2 (Table 1). For women, we found that #6, #1, and #2 were most relatable, for men #2, #3, and #1. Afghans found #2, #6, and #1 most relatable, while Iranians found #3, #11, and #6 most relatable.

**Table 1:** Person relatability ratings (0-5) for all participants, by Gender and by Nationality

	all participants					Gender									Correlations	
						Women			Men			Difference			Age	Distress
Person	N	M	Median	SD		M	SD		M	SD		t	p		rho	rho
Person #1	277	2.27	2	1.74		2.38	1.72		2.24	1.74		0.60	0.55		0.003	0.376⁎⁎⁎
Person #2	266	2.35	2	1.79		2.32	1.72		2.36	1.82		-0.20	0.84		0.012	0.214⁎⁎⁎
Person #3	259	2.25	2	1.91		2.23	1.92		2.26	1.92		-0.13	0.90		0.022	0.328⁎⁎⁎
Person #4	250	1.80	1	1.75		1.79	1.76		1.79	1.75		0.00	1.00		-0.053	0.250⁎⁎⁎
Person #5	244	1.81	1	1.72		2.04	1.77		1.73	1.69		1.25	0.21		-0.215⁎⁎⁎	0.142*
Person #6	239	2.36	2	1.85		2.83	1.90		2.18	1.80		2.42	0.02		-0.094	0.256⁎⁎⁎
Person #7	236	1.69	1	1.75		1.63	1.72		1.70	1.76		-0.28	0.78		-0.069	0.187⁎⁎
Person #8	229	1.86	1	1.75		2.08	1.76		1.80	1.75		1.06	0.29		-0.022	0.144*
Person #9	225	2.20	2	1.77		2.21	1.84		2.17	1.74		0.17	0.87		0.001	0.289⁎⁎⁎
Person #10	220	1.74	1	1.77		1.75	1.77		1.73	1.79		0.06	0.95		-0.043	0.242⁎⁎⁎
Person #11	215	2.16	2	1.77		2.19	1.92		2.12	1.71		0.25	0.81		0.013	0.246⁎⁎⁎

Note: M=Mean; Min=minimum score; Max=maximum score; SD=Standard Deviation

⁎ p<.05

⁎⁎ p<.01

⁎⁎⁎ p<.001

### Storyline relatability

We found a significant correlation between age and relatability of story #2 and story #3; and of distress with relatability of all stories except story #13 (Table 2). Across all participants, we found that the highest rated storylines were story #7, followed by story #8, and story #15 (Table 2). Compared with men, women related significantly stronger with story #2 (Table 2). Moreover, compared with Iranians, Afghans related significantly stronger with story #13 (Table 2). For women, we found that story #7, story #15, and story #8 were most relatable, for men story #7, story #8, and story #6. Afghans found story #7, story #8, and story #6 most relatable, while Iranians found story #7, story #15, and story #10 most relatable.

**Table 2:** Storyline relatability ratings (0-5) for all participants, by Gender and by Nationality

	all participants					Gender									Nationality			Correlations	
						Women			Men			Difference			Afghan			Age	Distress
Storyline	N	M	Median	SD		M	SD		M	SD		t	p		M	SD		rho	rho
Story #1	209	1.74	1	1.70		1.71	1.85		1.70	1.62		0.02	0.99		1.78	1.65		0.02	0.336⁎⁎⁎
Story #2	205	1.54	1	1.51		2.00	1.75		1.35	1.37		2.46	0.02		1.54	1.49		-0.06	0.282⁎⁎⁎
Story #3	200	1.77	1	1.62		1.85	1.61		1.71	1.61		0.54	0.59		1.73	1.57		0.194⁎⁎	0.279⁎⁎⁎
Story #4	200	1.77	1	1.62		1.85	1.61		1.71	1.61		0.54	0.59		1.73	1.57		0.194⁎⁎	0.279⁎⁎⁎
Story #5	195	1.35	1	1.57		1.19	1.52		1.37	1.57		-0.74	0.46		1.46	1.57		-0.10	0.228⁎⁎
Story #6	191	2.12	2	1.78		1.80	1.71		2.21	1.80		-1.43	0.16		2.13	1.70		0.00	0.236⁎⁎
Story #7	188	2.56	3	1.85		2.62	1.94		2.53	1.84		0.29	0.78		2.51	1.79		-0.06	0.351⁎⁎⁎
Story #8	185	2.31	2	1.83		2.15	1.79		2.36	1.86		-0.69	0.49		2.41	1.80		0.06	0.206⁎⁎
Story #9	183	1.38	1	1.56		1.27	1.51		1.39	1.57		-0.47	0.64		1.45	1.51		-0.07	0.181*
Story #10	182	1.79	1	1.79		1.67	1.81		1.84	1.79		-0.57	0.57		1.68	1.68		-0.01	0.369⁎⁎⁎
Story #11	176	1.64	1	1.64		1.63	1.66		1.64	1.65		-0.04	0.97		1.66	1.62		-0.04	0.243⁎⁎
Story #12	173	1.05	0	1.45		0.93	1.40		1.06	1.46		-0.53	0.60		1.06	1.40		-0.07	0.152*
Story #13	169	1.01	0	1.38		0.93	1.34		1.02	1.40		-0.35	0.73		1.13	1.41		-0.10	0.11
Story #14	166	1.36	1	1.59		1.54	1.76		1.29	1.54		0.86	0.39		1.37	1.55		0.03	0.221⁎⁎
Story #15	162	2.20	2	1.81		2.41	1.92		2.13	1.78		0.86	0.39		2.07	1.78		0.07	0.270⁎⁎⁎

Note: M=Mean; Min=minimum score; Max=maximum score; SD=Standard Deviation

⁎ p<.05

⁎⁎ p<.01

⁎⁎⁎ p<.001

### Additional feedback

The additional feedback that was provided by participants was not so much around the content testing process, but on the characters and storylines. This included suggestions that illustrations should portray a wider variety of ethnicities and ages. And, suggestions that background illustration should depict familiar surroundings and items; these included “landscape,” “carpets,” “kites,” and “writing in Farsi”. Some participants suggested a storyline that involved flashbacks depicting war in Afghanistan; others suggested a storyline where the character struggles to ask for help when something is wrong, determined to: “stay strong,” to the detriment of the character's mental health. These indicate that a number of respondents may be experiencing symptoms of PTSD from living in conflict zones. And that, help seeking behaviours are limited, likely due to cultural stigma and the connotation of being ‘weak’ if asking for help, which leads to a tendency to internalise and suppress needs.

## Discussion

Community and family are hugely important in both Iranian and Afghan culture, and men in particular play a prominent role.^17^ This is different to European countries like Switzerland, and so it was key that our characters and storyline reflect the unique experiences and the emotions and feelings felt by migrants. This population has a very particular experience, the culture and behaviours they have brought with them from their home country, the traumatic experiences they may have experienced en-route, and the countries and cultures they find themselves integrating into. The aim of this content testing was to adapt intervention materials in consideration of the significant issues faced by the Afghan / Persian migrants in Switzerland and other European host countries. With this study we aimed to identify the most relatable stories that can be used in the finalised intervention, however, we expected most if not all to be relatable to some extent. In the design of the characters and storylines, we made informed assumptions based on case examples from the clinical providers working with Afghan migrants in Switzerland. This team has worked with Afghan migrants in Switzerland providing psychological support through legal proceedings, mental health support and clinical evaluations for migrants in distress. Through this, they had engaged with migrants about their negative experiences and asked for their input on storylines, e.g. struggling with death or having financial pressures, and how those experiences make them think and feel. Identifying the emotions and feelings experienced by this community is crucial to providing adapted and tailored mental health services to Afghan / Persian refugees in host countries. Conducting content testing was an important step in the process of effectively adapting the START NOW program to treat symptoms of poor mental health in this population.

Incorporating characters and storylines with high scores of relatability into the program, ensures the content is symbolic of the participants' circumstances and they can recognise aspects of themselves and their lives. With this, participants are more likely to engage in sessions and reflect on the outcomes and reactions of the characters with empathy. When the content of a therapeutic program is culturally contextualised, it normalises mental health and the healing process, allows the participants to empathise to a greater extent, and therefore see the best results in a reduction of symptoms and improvement in mental health and wellbeing.

Across all participants the three characters with the highest scores of relatability were characters #6, #2, and #1, representing 1) feelings of guilt, 2) feelings of loneliness and 3) having no sense of purpose. This community's culture of collectivism, and individuals relying heavily on an extended network of family, may explain our results. Migrants often do not migrate with their entire immediate or extended family, and so feelings of guilt and loneliness are likely the result of experiencing migration and post-migration stressors with a much smaller network of support than they are typically accustomed to. Furthermore, when migrating, people will experience loss of a plethora of material and immaterial things, including a sense of purpose.

Future development of START NOW interventions should focus on addressing these feelings of guilt and loneliness and the mental health symptoms that are likely to result from these, such as depression or grief. These emotions and symptoms are not exclusive to migrants from Afghanistan or Iran, and are likely experienced by other refugees and asylum seekers. Thus, the characters and storylines developed for this content testing can be adapted to other cultures, and the START NOW intervention adapted to treat other populations from conflict settings.

The level of psychological distress experienced by participants only correlated with nationality, indicating that gender and age has little to no effect, but that levels of distress are associated with lived experiences and perception of those experiences. Why Iranian participants reported greater distress than Afghan participants needs further investigation, but it may be theorised that the former have higher levels of awareness of mental health, more advanced help seeking behaviours and a greater ability to externalise.

There is a clear correlation between reporting high levels of distress and higher relatability to characters, this however is to be expected, as the characters and storylines feature negative experiences that cause stress and likely resonate with many of the participants. Whilst we see no connection between gender and levels of stress, the results do show a difference in relatability to certain characters and storylines for men and women. To maximise the effectiveness of the intervention, it may be worth considering modifying the skills training - using different characters and storylines - for each gender. Another consideration when assigning groups should be age, as we found a significant correlation between age and reliability to certain characters and storylines. Again, this would allow for the most relatable characters and storylines to be featured in each group's skills training, and outcomes to be optimised.

We do see a pattern between wanting to remain in Switzerland and higher levels of distress; we can safely assume the lengthy and oftentimes complex asylum seeking procedure is likely to be the cause of a reasonable amount of that stress. In future studies, we want to evaluate how several factors surrounding the respondents' stay in Switzerland affects their experiences, and thereafter their relatability to characters and storylines. Variables such as length of stay, asylum decision received, when they arrived - and what political or social events or attitudes were prevalent at the time -, and to what extent they have integrated into Swiss society.

Content testing enables the adaptation of START NOW to be informed by the communities which it will treat. Next steps include developing the intervention sessions of the START NOW program, featuring the most relatable characters and storylines within them. In a randomised control trial (RCT), the efficacy of the intervention will be tested with migrants from Afghanistan and Iran now residing in Switzerland as participants. Whilst the trial will take place in Switzerland, the intervention is applicable to Persian / Afghan populations elsewhere. And, whilst this pilot study focuses on Persian / Afghan migrants as the target group, the material and intervention can be further adapted for migrant populations from other countries, with the results of the RCT expected to better inform these adaptations.

### Ethical statement

**Fig. 1:**
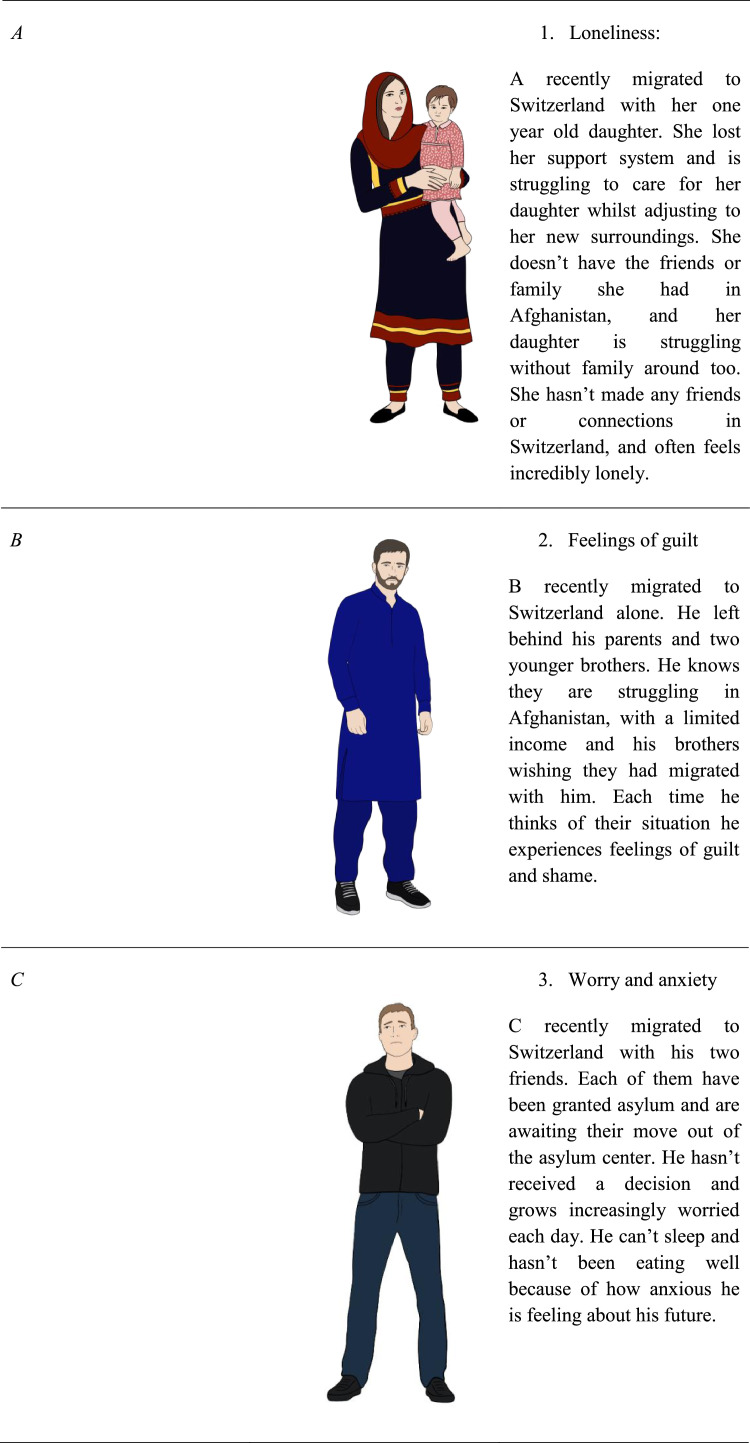

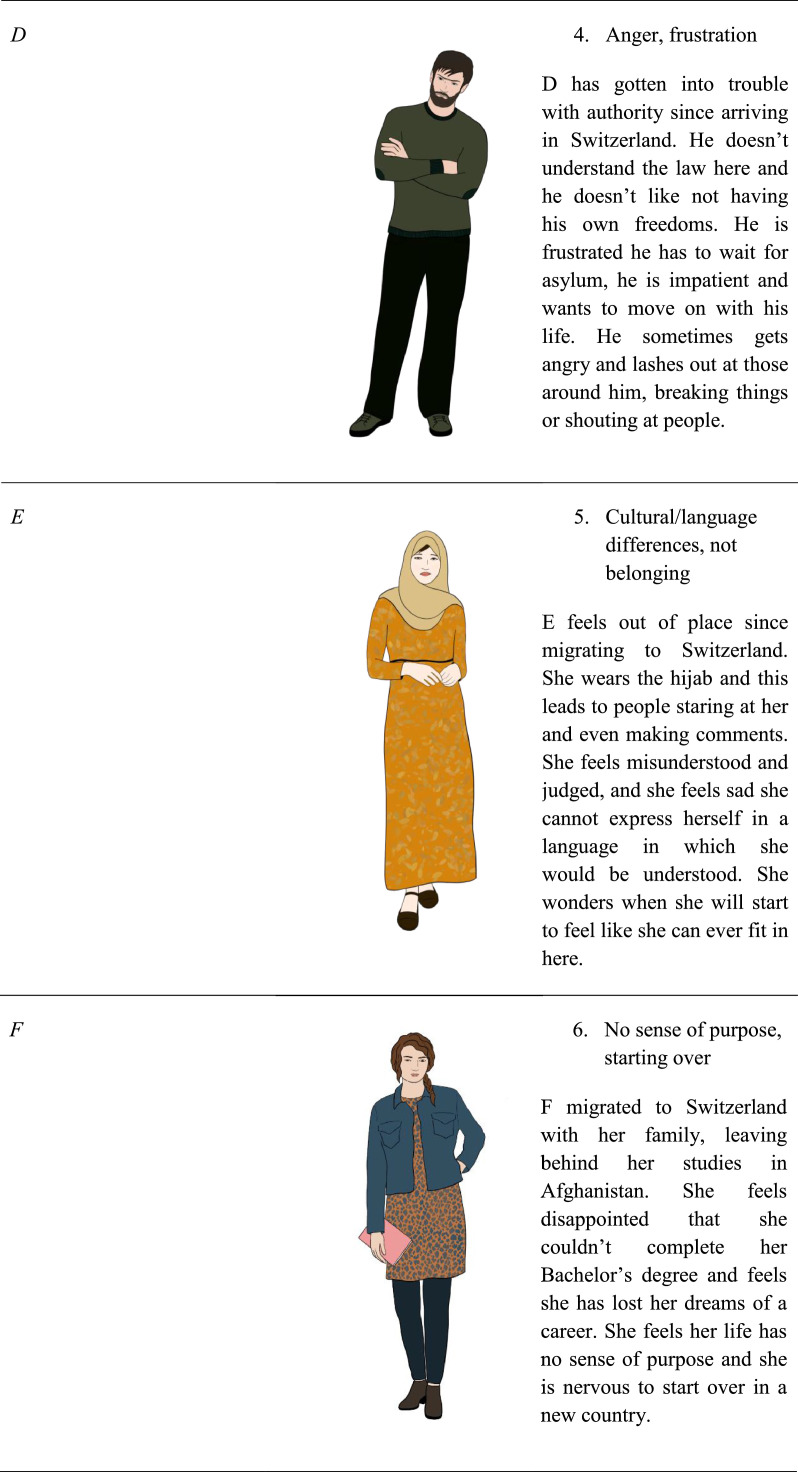

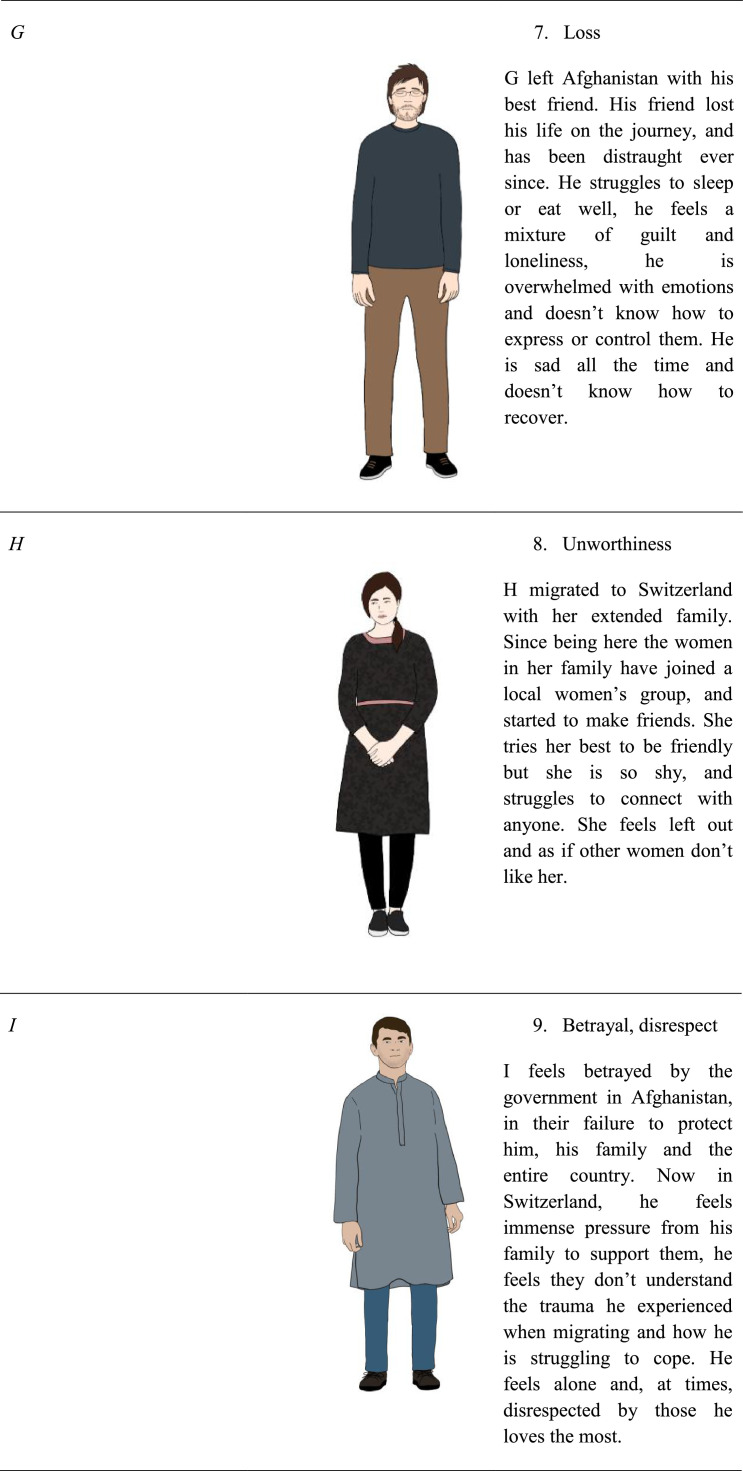

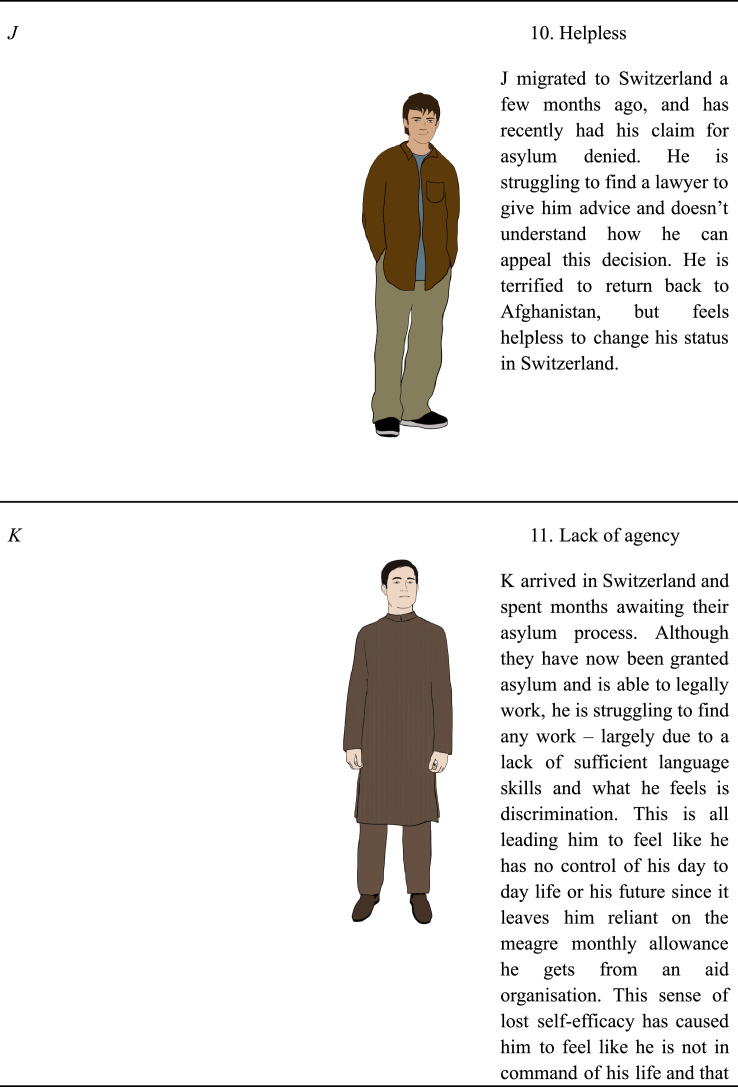
Characters and their description used in content testing.

**Fig. 2:**
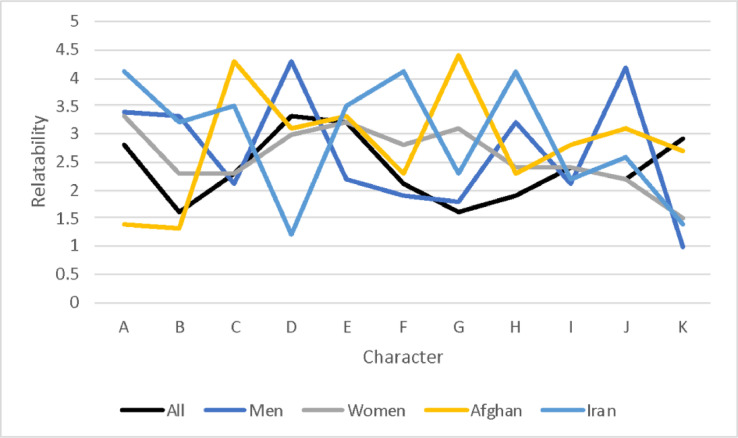
Character Relatability.

Insights may help improve current health promotion of migrants in Switzerland through adapting a feasible, skills training equipped to overcome the barriers to adequate care services. Ultimately, effects of START NOW on psychological health may facilitate positive life outcomes and decrease costs associated with treating migration- or conflict-related trauma. As such, participation in the present study can be considered ethical and fair, especially considering the large potential impact the present training may have on an individual as well as the wider migrant community.Table 3 Fig. 1Fig. 2

**Table 3:** Proportion of participant's relatability ratings per Person and Story

	Rating = 0	Rating = 1	Rating = 2	Rating = 3	Rating = 4	Rating = 5
Person #1	20.2	21.7	11.2	20.2	10.8	15.9
Person #2	21.8	17.7	12.0	17.7	13.5	17.3
Person #3	26.6	17.8	12.4	12.4	8.9	22.0
Person #4	32.8	22.4	8.8	16.0	8.0	12.0
Person #5	30.7	22.5	13.5	12.7	9.0	11.5
Person #6	23.4	15.9	13.8	16.3	9.2	21.3
Person #7	37.7	17.8	13.1	12.7	6.8	11.9
Person #8	29.3	22.7	14.8	13.1	5.7	14.4
Person #9	25.3	16.0	13.3	20.4	8.9	16.0
Person #10	36.8	18.6	11.4	11.8	10.0	11.4
Person #11	26.0	14.9	16.7	17.2	9.8	15.3
Story #1	34.4	19.1	12.4	16.7	6.7	10.5
Story #2	33.2	22.9	18.5	14.6	3.9	6.8
Story #3	29.5	21.0	18.5	13.5	9.0	8.5
Story #4	29.5	21.0	18.5	13.5	9.0	8.5
Story #5	44.6	19.5	10.8	11.8	8.2	5.1
Story #6	28.3	14.1	14.7	17.8	11.0	14.1
Story #7	19.1	17.6	11.2	15.4	13.3	23.4
Story #8	24.3	14.6	17.3	12.4	12.4	18.9
Story #9	41.0	23.0	12.6	8.7	9.8	4.9
Story #10	35.2	19.2	10.4	14.8	7.1	13.2
Story #11	33.5	24.4	11.4	14.2	8.0	8.5
Story #12	52.0	23.1	7.5	7.5	5.2	4.6
Story #13	51.5	22.5	11.8	7.1	2.4	4.7
Story #14	44.0	19.3	14.5	9.0	6.0	7.2
Story #15	26.5	13.0	19.1	14.8	8.6	17.9

## CRediT authorship contribution statement

**Lyla Schwartz:** Conceptualization, Data curation, Formal analysis, Investigation, Methodology, Project administration, Visualization, Writing – original draft, Writing – review & editing. **Donja Brunner:** Formal analysis, Software, Supervision, Validation, Writing – review & editing. **Eva Unternährer:** Formal analysis, Software, Supervision, Writing – review & editing. **Christina Stadler:** Funding acquisition, Supervision, Validation, Writing – review & editing.

## Footnotes

4 Schengenvisa News. (2022). Asylum Applications in Switzerland Drop by 9.8% in February – Afghanis Remain Leading National Group of Applicants.

5 Maier, T., Schmidt, M., Mueller, J. (2010). Mental health and healthcare utilization in adult asylum seekers. *Swiss Med Wkly.*

6 Ibid.

7 Ibid.

8 Marrone, S. (2007). Understanding barriers to health care: a review of disparities in health care services among indigneous populations. *International Journal of Circumpolar Health.*

9 Ibid.

10 Benish, S.G. (2011). Culturally adapted psychotherapy and the legitimacy of myth: a direct-comparison meta analysis. *Journal of Counselling Psychology.*

11 Resnicow, K. (1999). Cultural sensitivity in public health: defined and demystified. *Ethnicity & Disease.*

12 Heim, E. Kohrt, B. A. (2019). Cultural Adaptation of Scalable Psychological Interventions: A New Conceptual Framework. *Clinical Psychology in Europe.*

13 https://www.carilionclinic.org/start_now#about

14 https://www.istartnow.ch/de/

15 Ibid

16 Stadler, C. et al. (2016). START NOW: A cognitive behavioral skills training for adolescent girls with conduct disorder and oppositional defiant disorder - A randomized clinical trial. *Lancet Psychiatry.*

17 Echavez, C. R., Mosawi S. M., Pilongo, L. (2016). The Other Side of Gender Inequality: Men and Masculinities in Afghanistan. *Afghanistan Research and Evaluation Unit.*

